# The C825T Polymorphism of the G-Protein β3 Subunit Gene and Its Association with Hypertension and Stroke: An Updated Meta-Analysis

**DOI:** 10.1371/journal.pone.0065863

**Published:** 2013-06-14

**Authors:** Lu Guo, Li-Li Zhang, Bo Zheng, Yun Liu, Xiao-Jie Cao, Yan Pi, Bing-Hu Li, Jing-Cheng Li

**Affiliations:** 1 Department of Neurology, Institute of Surgery Research, Daping Hospital, Third Military Medical University, Yuzhong District, Chongqing, PR China; 2 Department of Neurology, Southwest Hospital, Third Military Medical University, Shapingba District, Chongqing, PR China; Vanderbilt University Medical Center, United States of America

## Abstract

**Objective:**

Several epidemiological studies have evaluated the association between the *GNB3* C825T polymorphism and hypertension or stroke. The results of these studies were inconsistent; therefore, we performed a meta-analysis to clarify these discrepancies.

**Methods:**

We systematically searched the PubMed, Embase, Web of Science, CNKI, and CBM databases, and manually searched reference lists of relevant papers, meeting abstracts, and relevant journals. Pooled odds ratios (ORs) and 95% confidence intervals (CIs) were calculated for dominant, recessive, and allelic models. A fixed or random effects model was separately adopted depending on study heterogeneity. Subgroup and sensitivity analyses were performed to detect study heterogeneity and examine result stability, respectively. Publication bias was tested using funnel plots, the Egger's regression test, and Begg's test.

**Results:**

We screened 66 studies regarding hypertension and eight concerning stroke. A combined analysis showed that only the allelic model found a marginal association with hypertension (OR = 1.07, 95% CI = 1.01–1.13) and female gender (OR = 1.11, 95% CI = 0.99–1.24). However, no comparison models found an association with stroke (allelic model: OR = 1.11, 95% CI = 0.94–1.32; dominant model: OR = 1.16, 95% CI = 0.92–1.48; and recessive model: OR = 1.05, 95% CI = 0.97–1.14). Sensitivity analysis suggested that all models did not yield a relationship to hypertension or stroke among Asians. Besides, there was a lack of statistical association with hypertension in Caucasians, which maybe due to a small sample size. When we restricted the included studies to normal populations according to the Hardy–Weinberg equilibrium, no association was found.

**Conclusions:**

There was no evidence indicating that the 825T allele or TT genotype was associated with hypertension or stroke in Asians or hypertension in Caucasians. However, further studies regarding Africans and other ethnicities are needed to identify further correlations.

## Introduction

Hypertension is a major risk factor of stroke, cardiovascular disease, and end-stage renal disease and affects about 1 billion adults worldwide, including 3.8 million in Taiwan and 160 million in China [1]. Stroke is a primary contributor to long-term adult disability and the third most common cause of death in developed countries [2], [3]. Blood pressure-lowering therapies are viewed as protective measures against the risk of hypertension and stroke, but both genetic and lifestyle factors are likely involved in the development of these conditions.

Guanine nucleotide-binding proteins (G proteins) are key determinants of specific and temporal characteristics of many signaling processes and are expressed in all cells of the human body to primarily transduce signals from the cell surface into a cellular response. G proteins consist of α, β, and γ subunits and different genes encode for 18 α subunits, 5 β subunits, and 12 γ subunits, which enable the formation of highly variable heterotrimers [4]. Activation of a G protein-coupled receptor results in an exchange of guanosine triphosphate for guanosine diphosphate followed by dissociation of the α subunit from the βγ complex. Different α subunits can then regulate a large variety of intracellular signaling cascades. The α subunit and βγ complex then reassemble as a heterotrimer available for a new activation cycle [5]. Reportedly, the α, β, γ subunit composition of G proteins determine the receptor and effector specificities of particular heterotrimers. Thus, alterations in G protein signaling can cause multiple disorders and it is likely that functionally important genetic polymorphisms in genes that encode human G protein subunits can cause or contribute to various disease phenotypes.

The G protein beta polypeptide 3 (*GNB3*) gene encodes the Gβ3 subunit of heterotrimeric G proteins and is located on chromosome 12p13 and comprises 11 exons and 10 introns. A polymorphism (C825T, rs5433) was found to be associated with a shortened splice variant of the Gβ3 protein that gives rise to enhanced signal transduction via pertussis toxin-sensitive G proteins [6], [7]. The C825T polymorphism located in exon 10 is in close linkage disequilibrium with the A(-350)G promoter single nucleotide polymorphism (SNP) and the C1429T SNP and can serve as a marker for allele-specific GNB3 expression. However, differential G protein activities associated with the C825T SNP did not result from different transcript amounts associated with specific GNB3 genotypes [8].

Several epidemiological studies have shown an association between the *GNB3* 825T allele and other features of metabolic syndrome, including obesity, insulin resistance, changes in autonomic nervous function, and dyslipidemia. This polymorphism has also been identified in hypertension, stroke, Alzheimer’s disease, sudden death, tumor progression, and as a genetic marker for drug responses to diuretics, antidepressants, and the antihypertension medications sildenafil, clonidine, and sibutramine [9]–[12].

Recently, many groups have investigated the relationship between the *GNB3* C825T polymorphism and hypertension or stroke; however, the results have been inconclusive. Therefore, we designed the present meta-analysis to better clarify the association between the *GNB3* C825T polymorphism and hypertension or stroke.

## Materials and Methods

### Literature Search

This meta-analysis followed the PRISMA (preferred reporting items for systematic reviews and meta-analyses) criteria [13]. We comprehensively searched for related papers in the following electronic databases: PubMed (up to Nov 2012), Embase (1996 to Nov 2012), Web of Science (2003 to Nov 2012), CBM (China Biology Medicine, 1978 to Jul 2012) and CNKI (China National Knowledge Infrastructure, 1999 to Nov 2012) using various keywords, including “hypertension,” “stroke,” “cerebral hemorrhage,” “cerebrovascular disorder,” “cerebrovascular disease,” “mutation,” “variant,” “polymorphism,” “ischemic stroke,” “GNB3,” “G protein beta,” and “G-beta.” Then, we manually searched the relevant journals and co-authors listed in the included studies to find additional studies. Reference lists of all retrieved publications were also checked for missing information. Meeting abstracts, which were previously shown to influence meta-analytical results [14], were also scrutinized. All relevant articles were initially scanned on the basis of title, keywords, and abstract. If this was not possible, the full text was obtained for further evaluation. The literature retrieval was performed independently by three investigators (LG, LLZ, and BZ) and discrepancies were resolved by reaching a consensus among the investigators. If a consensus could not be established, a fourth reviewer (JCL) was consulted to resolve the discrepancy. The last database searches were performed on November 10, 2012.

### Inclusion Criteria

Studies were screened that met the following criteria: (1) population-based or hospital-based case-control studies regarding the relationship between the *GNB3* C825T polymorphism and essential hypertension or stroke; (2) sufficient data on genotypic and allelic frequencies to determine an odds ratio (OR) with a 95% confidence interval (CI). If multiple publications reported the same or overlapping data, the most recent or complete study or the largest population was included in this meta-analysis as described by Little et al. [15]; (3) to avoid local literature bias, publications in both Chinese and English were considered [16]; (4) studies with related clinical characteristics were limited to those using human subjects; (5) articles regarding cases compounded with other diseases, such as diabetes mellitus and myocardial infarction, were also included; and (6) if patient blood pressure was measured casually or ambulatory (24 h), the latter were used. Hypertension was defined as mean casual blood pressure ≥140/90 mmHg or mean ambulatory blood pressure >134/79 mmHg.

### Data Extraction

Data were independently extracted from each study by three investigators (LG, LLZ, and BZ) following the above-mentioned inclusion criteria. Discordance was resolved by discussion or another reviewer (JCL) was consulted. The following data were collected from each of the selected studies: surname of the first author, year of publication, country of origin, population ethnicity, source of control, T allele frequency in controls, genotype variance in the cases and controls, and the Hardy–Weinberg equilibrium (HWE) using the χ^2^ test. A *p*-value of <0.05 for the HWE was considered statistically significant.

### Quality Score Assessment

The quality of each selected study was assessed independently by the same three investigators according to the Newcastle–Ottawa Scale (NOS) (www.ohri.ca/programs/clinical_epidemiology/oxford.asp). Scores were based on the selection, comparability, and exposure (case-control studies) or outcome (cohort studies) of the studies. To avoid selection bias, studies of poor quality were not rejected in this meta-analysis.

### Statistical Analysis

All statistical analyses were conducted using Stata statistical software ver. 11.0 (Stats Corp., College Station, TX, USA) and Review Manager ver. 5.0 (The Cochrane Collaboration, Oxford, UK). All tests were two-sided and a *p*-value <0.05 was considered statistically significant. The strength of association of the *GNB3* C825T polymorphism with hypertension or stroke was measured by calculating summary ORs with corresponding 95% CIs for the dominant model (TT+CT vs. CC), recessive model (TT vs. CT+CC), and allelic model (T allele vs. C allele), respectively.

Heterogeneity between the studies was analyzed using the Cochran’s Q test and the I^2^ statistic (range, 0–100%) [17], [18]. If the results of the Q test was *p*<0.1 and the measure of I^2^ was >50%, indicating significant heterogeneity between studies, the ORs were pooled using a fixed effects Mantel–Haenszel method [19], otherwise the DerSimonian and Laird random effects model was adopted [20], [21]. A Galbraith plot was employed to detect potential sources of heterogeneity [22]. The pooled ORs were recalculated after removing outlier studies identified by the Galbraith plots. To further detect heterogeneity, subgroup analyses were performed using the status of the HWE (yes or no) or the control source.

Sensitivity analysis was conducted by limiting the meta-analysis to high quality studies (NOS score ≥8). We also performed the analyses a second time by limiting the studies according to the HWE and excluding those that included myocardial infarction, obesity, or diabetes mellitus in the cases or controls. Sensitivity analysis was performed to identify alterations in the overall significance of the estimate.

Cumulative meta-analysis was performed to identify the influence of the first published study on the subsequent publications concerning the relationship between the *GNB3* C825T polymorphism and hypertension, and to estimate the combined estimate over time [23].

Publication bias was assessed using the Egger's regression test and Begg's test. The Egger’s test detects funnel plot asymmetry by determining whether the intercept deviates significantly from zero in a regression of the standardized effect estimates against their precision [24], [25]. These methods were based on plotting the estimate (logOR) against the corresponding standard error (SE).

## Results

### Study Selection and Characteristics

**Figure 1:**
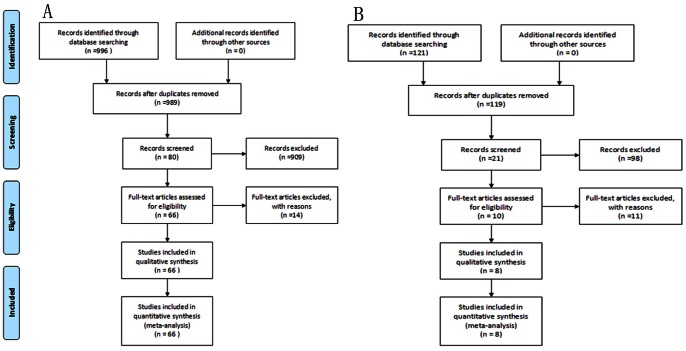
A flow diagram of the literature search for associations between the *GNB3* C825T polymorphism and hypertension (A) or stroke (B).

The present study met the PRISMA statement requirements (Appendix S1). Through comprehensive retrieval and evaluation, 66 studies (20,782 cases and 26,141 controls) regarding hypertension and eight studies (3,427 cases and 3,948 controls) regarding stroke met the inclusion criteria and were included in the final meta-analysis. Details of the included studies are presented in Tables 1 and 2 and the selection process is shown in Figure 1.

**Table 1:** The main characteristics of included studies regarding the association between the *GNB3* C825T polymorphism and hypertension.

Author	Year	Country	Ethnicity	Sample sizeHT/Control, n	HT/Control, n			HT/Control, n		HEWY/N	T frequencyin control	SOCPB/HB	Score
					CC	CT	TT	C	T				
rand [52]	2003	Belgian	Caucasian	352/1160	173/542	151/511	28/107	497/1595	207/725	Y	0.313	PB	9
Shioji [53]	2003	Japan	Asian	775/1105	177/287	385/536	213/282	739/1110	811/1100	Y	0.498	PB	8
Dong [54]	1999	London	African	185/243	3/14	61/83	121/146	67/111	303/375	Y	0.772	PB	8
Yamamoto [55]	2004	Japan	Asian	266/540	70/162	120/239	76/139	260/563	272/517	N	0.479	PB	8
Hayakawa [56]	2007	Japan	Asian	156/271	42/82	76/121	38/68	160/285	152/257	Y	0.474	HB	9
Khamidullaeva [57]	2011	Uzbek	Asian	174/60	64/0	93/50	17/10	221/50	127/70	Y	0.583	PB	9
Hui [58]	2007	Japan	Asian	261/271	78/72	115/148	68/51	271/292	251/250	Y	0.461	PB	9
Alioglu [59]	2008	Turkey	Asian	209/82	37/27	124/40	48/15	198/94	220/70	Y	0.427	PB	8
Kato [31]	1998	Japan	Asian	718/515	187/128	359/263	172/124	733/519	703/511	Y	0.496	PB	9
Tsai [60]	2000	China	Asian	302/199	57/43	149/96	96/60	263/182	341/216	Y	0.543	PB	9
Kedzierska [61]	2006	Poland	Caucasian	26/18	9/15	12/2	5/1	30/32	22/4	Y	0.111	HB	9
Hager [62]	2011	Finland	Caucasian	74/48	32/24	27/19	15/5	91/67	57/29	Y	0.302	HB	8
Marcun Varda [63]	2006	Slovenia	Caucasian	104/200	53/104	42/80	9/16	148/288	60/112	Y	0.280	HB	9
Holmen [64]	2010	Norway	Caucasian	1661/1175	863/630	682/465	116/80	2408/1725	914/625	Y	0.266	PB	9
Tozawa [65]	2001	Japan	Asian	179/180	32/39	68/82	79/59	132/160	226/200	Y	0.556	HB	8
Wang [66]	2004	Kazak	Asian	264/244	76/67	129/119	59/58	281/253	247/235	Y	0.482	PB	8
Buchmayer [67]	2000	Australia	Caucasian	174/174	85/72	70/85	19/17	240/229	108/119	Y	0.342	PB	8
Yamagishi [68]	2006	Japan	Asian	640/792	159/156	321/415	160/221	639/727	641/857	Y	0.541	PB	9
Beige [69]	1999	Germany	Caucasian	479/900	204/514	224/312	51/74	632/1340	326/460	N	0.256	PB	9
Zychma [70]	2000	Poland	Caucasian	85/68	32/24	44/36	9/8	108/84	62/52	Y	0.382	PB	8
Benjafieid [41]	1997	Australia	Caucasian	110/189	27/101	71/82	12/6	125/284	95/94	N	0.249	PB	8
Li(a) [71]	2005	China	Asian	501/503	142/137	256/259	103/107	540/533	462/473	Y	0.470	PB	8
Suwazono [51]	2006	Japan	Asian	218/1052	47/345	121/719	50/288	215/1409	221/1295	N	0.479	PB	9
Ishikawa(a) [26]	2000	Japan	Asian	304/422	43/37	90/85	48/43	184/159	186/171	Y	0.518	HB	9
Ishikawa(b) [26]	2000	Japan	Asian	181/165	67/96	161/204	76/122	295/396	313/448	Y	0.531	HB	9
Bae [72]	2007	Korea	Asian	687/924	193/217	319/469	175/238	705/903	669/945	Y	0.511	PB	9
Panoulas [73]	2009	Britain	Caucasian	269/114	128/50	113/54	28/10	369/154	169/74	Y	0.325	HB	8
Huang [74]	2003	China	Asian	585/580	134/126	290/303	161/151	558/555	612/605	Y	0.522	PB	9
Larson [75]	2000	America	African	472/432	29/25	190/170	253/237	248/220	696/644	Y	0.745	PB	8
Suwazono [76]	2004	Japan	Asian	332/2289	78/574	171/1216	83/499	327/2364	337/2214	N	0.484	PB	8
Brand [77]	1999	France/Ireland	Caucasian	206/467	98/226	92/197	16/44	288/649	124/285	Y	0.305	PB	8
Nejatizadeh [78]	2011	Iran	Asian	449/345	185/192	211/144	53/9	581/528	317/162	N	0.235	PB	9
Pitsavos [79]	2006	Greece	Caucasian	136/239	65/126	60/86	11/27	190/338	82/140	N	0.293	PB	8
Izawa [80]	2003	Japan	Asian	574/533	138/159	291/261	145/113	567/579	581/487	Y	0.457	PB	9
Ozkececi [81]	2008	Turkey	Asian	99/45	35/26	51/15	13/4	121/67	77/23	Y	0.256	PB	8
Yin [82]	2009	China	Asian	257/865	60/224	126/424	71/217	246/872	268/858	Y	0.496	PB	9
Vasudevan [32]	2009	Malaysian	Asian	70/75	19/20	32/44	19/11	70/84	70/66	Y	0.440	PB	8
Dong [83]	2006	China	Asian	97/87	25/27	47/46	25/14	97/100	97/74	Y	0.425	PB	7
Zhang [84]	2007	China	Asian	143/124	68/54	59/58	16/12	195/166	91/82	Y	0.331	PB	8
Li(b) [85]	2005	China	Asian	321/147	92/40	167/69	62/38	351/149	291/145	Y	0.493	PB	8
Hu [86]	2006	China	Asian	135/124	60/54	59/58	16/12	179/166	91/82	Y	0.331	PB	7
Gai [87]	2007	China	Asian	136/197	31/54	73/95	32/48	135/203	137/191	Y	0.485	PB	7
Chen [88]	2007	China	Asian	109/378	25/104	52/219	32/55	102/427	116/329	N	0.435	PB	7
Tan(b) [89]	2003	China	Asian	112/112	38/66	60/40	14/6	136/172	88/52	Y	0.232	PB	7
Zhang [90]	2005	China	Asian	111/150	32/51	52/72	27/27	116/174	106/126	Y	0.856	PB	7
You [91]	2000	China	Asian	98/110	25/31	47/52	26/27	97/114	99/106	Y	0.482	PB	7
Jing [92]	2006	China	Asian	354/384	96/106	152/163	106/115	344/375	364/393	N	0.512	PB	8
Sun [93]	2003	China	Asian	117/151	41/51	56/78	20/22	138/180	96/122	Y	0.404	PB	7
Zhang [94]	2001	China	Asian	146/79	36/18	101/50	9/11	173/86	119/72	N	0.456	PB	8
Dou [42]	2009	Japan	Asian	2092/2810	480/679	1081/1380	531/751	2041/2738	2143/2882	Y	0.513	PB	9
Song (a) [27]	2011	China	Asian	122/104	17/26	78/49	27/29	112/101	132/107	Y	0.514	PB	9
Song (b) [27]	2011	China	Asian	102/92	34/18	40/43	28/31	108/79	96/105	Y	0.571	PB	9
Liu [95]	2009	China	Asian	269/229	93/67	106/100	70/62	292/234	246/224	Y	0.489	PB	8
Huang (a) [28]	2005	China	Asian	96/87	18/20	57/47	21/20	93/87	99/87	Y	0.500	PB	8
Huang (b) [28]	2005	China	Asian	34/151	9/37	21/97	4/17	39/171	29/131	N	0.434	PB	8
Lu [96]	2009	China	Asian	162/180	48/52	94/101	20/27	190/205	134/155	Y	0.431	PB	7
Li(c) [97]	2005	China	Asian	310/151	89/42	161/70	60/39	339/154	281/148	Y	0.490	PB	8
Zhao [98]	2009	China	Asian	331/293	117/52	179/137	35/104	413/241	249/345	Y	0.589	PB	7
Wang [99]	2011	China	Asian	92/110	30/34	50/70	12/6	110/138	74/82	N	0.373	PB	7
Wang [100]	2003	China	Asian	408/140	131/39	182/66	95/35	444/144	372/136	Y	0.486	PB	7
Li (a) [101]	2006	China	Asian	334/267	59/54	149/113	126/100	267/221	401/313	N	0.586	PB	8
Huang [102]	2007	China	Asian	502/489	142/135	257/252	103/102	541/522	463/456	Y	0.466	PB	8
Li (b) [103]	2006	China	Asian	268/218	47/48	132/85	89/85	226/181	310/255	N	0.585	PB	7
Dai [104]	2002	China	Asian	133/257	28/70	73/127	32/60	129/267	137/247	Y	0.481	PB	7
Zhang [105]	2006	China	Asian	100/100	19/32	46/53	35/15	84/117	116/83	Y	0.415	PB	7
Yang [106]	2007	China	Asian	170/196	53/60	98/118	19/18	204/238	136/154	N	0.393	PB	8
Li [39]	2003	China	Asian	641/370	119/85	313/157	209/128	551/327	731/413	N	0.558	PB	8
Liu [107]	2003	China	Asian	163/339	50/125	79/157	34/57	179/407	147/271	Y	0.400	PB	8
Tan(a) [108]	2003	China	Asian	40/31	11/14	25/15	4/2	47/43	33/19	Y	0.306	HB	7

HT, hypertension; SOC, source of control; PB, population-based, controls were blood donors, healthy controls matched for age, gender and domicile and participants in an health service programme from the same geographical region without clinically detectable hypertension; HB, hospital-based, controls were patients admitted to hospital without hypertension matched for age, gender and domicile; HWE, Hardy–Weinberg equilibrium; and MAF, minor allele frequency; Three publications [26]–[28] contained more than one independent population, therefore, we considered them as different studies. Two studies [57], [80] were limited to the relationship in males. The samples [54], [75] were from individuals of African descent.

**Table 2:** The main characteristics of the included studies regarding association between the *GNB3* C825T polymorphism and stroke.

Author	Year	Country	Ethnicity	Sample sizeStroke/Control, n	Stroke/Control, n			Stroke/Control, n		HEWY/N	T frequency in control	SOCPB/HB	Score
					CC	CT	TT	C	T				
Zhang [12]	2005	China	Asian	922/1124	212/244	512/569	198/311	936/1057	908/1191	Y	0.530	PB	8
Morrison [11]	2001	America	Caucasian	990/1124	266/311	512/569	212/244	1044/1191	936/1057	Y	0.470	PB	9
Zhao [34]	2001	China	Asian	294/280	89/93	144/133	61/54	322/319	266/241	Y	0.430	PB	7
Tan [35]	2003	China	Asian	100/100	32/65	58/32	10/3	122/162	78/38	Y	0.190	PB	7
Wang [36]	2011	China	Asian	80/110	26/34	46/70	8/6	98/138	62/82	N	0.373	PB	7
Zhao [38]	2000	China	Asian	715/668	196/195	348/338	171/135	740/728	690/608	Y	0.455	PB	8
Li [39]	2003	China	Asian	144/352	36/64	70/175	38/113	142/303	146/401	Y	0.570	PB	7
Zhao [37]	2004	China	Asian	182/190	35/55	87/92	60/43	157/202	207/178	Y	0.468	PB	7

HT, hypertension; SOC, source of control; PB, population-based, controls were blood donors, healthy controls matched for age, gender and domicile and participants in an health service programme from the same geographical region without clinically detectable hypertension; HB, hospital-based, controls were patients admitted to hospital without hypertension matched for age, gender and domicile; HWE, Hardy–Weinberg equilibrium; and MAF, minor allele frequency. Five studies [11], [12], [34]–[36] regarding the association of the GNB3 C825T polymorphism and ischemic stroke were identified while one [37] was regarding cerebral hemorrhage and the other two [38], [39] included ischemic stroke or cerebral hemorrhage cases.

Of the 66 studies, eight compared males and females to assess an association between the *GNB3* C825T polymorphism and hypertension. Among these articles, three publications [26]–[28] contained more than one independent population, and thus, we considered them as different studies that should be counted twice. Two studies [29], [30], which did not supply all of the required information regarding case or control genotypes were excluded from this meta-analysis. We only retrieved information on hypertensive patients and controls without diabetes mellitus from three studies [31]–[33]. Five studies [11], [12], [34]–[36] regarding the association of the *GNB3* C825T polymorphism and ischemic stroke were identified while one [37] was regarding cerebral hemorrhage and the other two [38], [39] included ischemic stroke or cerebral hemorrhage cases.

All of the included studies were case-controlled in design. The main characteristics of the included studies are summarized in Tables 1–3. In all of the included studies, genotyping was analyzed via polymerase chain reaction and restriction fragment length polymorphisms. Stroke cases were evaluated by strict neurological examination: computed tomography, nuclear magnetic resonance imaging or both.

**Table 3:** The association between the *GNB3* C825T polymorphism and hypertension among males and females.

				Male(HT/Control),n					Female(HT/Control),n				
Author	Year	Country	Ethnicity	CC	CT	TT	C	T	CC	CT	TT	C	T
Khamidullaeva [57]	2011	Uzbek	Asian	64/0	93/50	17/10	221/50	127/70	Not available				
Hui [58]	2007	Japan	Asian	57/50	69/100	44/32	183/200	157/164	21/22	46/48	24/19	88/101	94/117
Tsai [60]	2000	China	Asian	28/21	70/39	30/30	126/81	130/99	29/22	79/57	58/30	137/101	195/117
Holmen [64]	2010	Norway	Caucasian	404/245	340/194	58/41	1148/684	456/276	459/385	340/271	58/39	1258/1041	456/349
Buchmayer [67]	2000	Australia	Caucasian	40/33	36/43	11/11	116/109	58/65	45/39	34/42	8/6	124/120	50/54
Suwazono [51]	2006	Japan	Asian	35/180	90/372	30/171	160/732	150/714	12/165	31/347	20/117	55/677	71/581
Suwazono [76]	2004	Japan	Asian	58/300	135/614	63/282	251/1214	261/1178	20/274	36/602	20/217	76/1150	76/1036
Izawa [80]	2003	Japan	Asian	138/159	291/261	145/113	567/579	581/487	Not available				

HT, hypertension.

### Quantitative Synthesis

**Figure 2:**
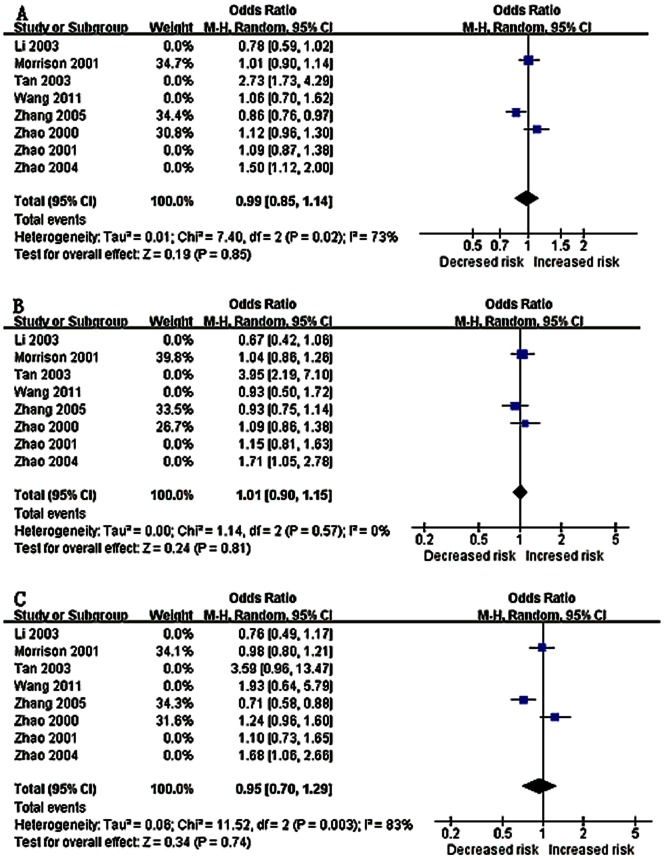
A forest plot for (A) the allelic model (T allele vs. C allele), (B) the dominant model (GG+GA vs. AA), and (C) the recessive model (TT vs. CT+CC). Random effects models were used with I^2^ values of 81, 76, and 71%. No evidence of association between the *GNB3* C825T polymorphism and stroke were detected in the allelic model (OR = 1.11, 95% CI = 0.94–1.32), dominant model (OR = 1.16, 95% CI = 0.92–1.48), or recessive model (OR = 1.08, 95% CI = 0.84–1.38).

All models concerning the association of the *GNB3* C825T polymorphism and hypertension or stroke were identified using the random effects model for I^2^>50%, which suggested significant heterogeneity. However, in most of the models, I^2^ was ≥70%, which indicated high heterogeneity [40], thus we pooled the ORs because of the significant results. The main results of this meta-analysis are presented in Tables 4 and 5. A significant overall association between the *GNB3* C825T polymorphism and the risk of hypertension was only detected in the allelic model (OR = 1.07, 95% CI = 1.01–1.13). No evidence of significance was identified in the dominant model (OR = 1.08, 95% CI = 0.98–1.81) or the recessive model (OR = 1.05, 95% CI = 0.97–1.14). However, none of the comparison models found an association between the *GNB3* C825T polymorphism and stroke (allelic model: OR = 1.11, 95% CI = 0.94–1.32; dominant model: OR = 1.16, 95% CI = 0.92–1.48; and recessive model: OR = 1.05, 95% CI = 0.97–1.14, respectively) (Figure 2). After excluding the outlier studies identified by the Galbraith plots, heterogeneity was effectively nonexistent or decreased and the pooled ORs were similar to those when the outlier studies regarding stroke cases were included; however, the association to hypertension was significant using the dominant model (OR = 1.05, 95% CI = 1.00–1.11). These results suggested that carriers of the T allele or TT genotype may have a higher risk of hypertension than non-carriers; however, the *GNB3* C825T polymorphism was not a risk factor for stroke.

**Table 4:** The main results of meta-analysis of the association between the *GNB3* C825T polymorphism and hypertension.

	T allele vs. C allele (allelic model)			TT+CT vs. CC (dominant model)			TT vs. CT+CC (recessive model)		
Study group	OR (95%CI)	*p*	I ^2^	OR (95%CI)	*p*	I^2^	OR (95%CI)	*p*	I^2^
Overall	1.07 (1.01,1.13)	0.02	71%	1.08 (0.98,1.81)	0.11	74%	1.05 (0.97,1.14)	0.23	58%
Excluding outlier studies	1.03 (1.00,1.06)	0.06	0%	1.05 (1.00,1.11)	0.03	0%	1.00 (0.95,1.05)	0.92	0%
Male	0.93 (0.79,1.11)	0.43	71%	1.01 (0.80,1.28)	0.92	59%	1.02 (0.87,1.18)	0.82	45%
Female	1.11 (0.99,1.24)	0.08	0%	1.05 (0.90,1.24)	0.53	0%	1.35 (1.07,1.70)	0.01	0%
Caucasian	1.18 (1.00,1.39)	0.05	76%	1.22 (0.97,1.54)	0.09	79%	1.10 (0.90,1.34)	0.36	20%
Asian	1.05 (0.99,1.11)	0.12	68%	1.05 (0.94,1.16)	0.39	72%	1.04 (0.95,1.15)	0.37	63%
HWE									
Y	1.03 (0.97,1.10)	0.32	68%	1.04 (0.96,1.14)	0.34	58%	1.02 (0.93,1.11)	0.71	52%
N	1.18 (1.06,1.33)	0.004	70%	1.13 (0.86,1.48)	0.39	88%	1.19 (0.96,1.47)	0.11	71%
Source of control									
HB	1.07 (0.99,1.16)	0.07	0%	1.15 (0.92,1.44)	0.23	35%	1.11 (0.89,1.39)	0.34	17%
PB	1.07 (1.00,1.13)	0.05	74%	1.07 (0.97,1.18)	0.21	76%	1.04 (0.96,1.14)	0.34	62%
Normal population*	1.04 (0.97,1.12)	0.25	69%	1.05 (0.96,1.16)	0.30	61%	1.03 (0.93,1.14)	0.57	59%
Score≥8	1.08 (1.01,1.16)	0.03	76%	1.07 (0.97,1.18)	0.20	75%	1.03 (0.97,1.11)	0.34	32%

*p*, a *p*-value of combined effect; CI, confidence interval;

* , We conducted the analyses by limiting the studies according to the HWE and excluding those that included myocardial infarction, obesity, or diabetes mellitus in the cases or controls.

**Table 5:** The main results of meta-analysis of association between the *GNB3* C825T polymorphism and stroke.

	T allele vs. C allele (allelic model)			TT+CT vs. CC (dominant model)			TT vs. CT+CC (recessive model)		
Study group	OR (95%CI)	*p*	I ^2^	OR (95%CI)	*P*	I^2^	OR (95%CI)	*p*	I^2^
Overall	1.11 (0.94,1.32)	0.22	81%	1.16 (0.92,1.48)	0.21	76%	1.08 (0.84,1.38)	0.54	71%
Excluding outlier studies	1.06 (0.97,1.15)	0.20	0%	1.05 (0.94,1.17)	0.36	13%	1.11 (0.96,1.29)	0.16	34%
Asian	1.15 (0.92,1.43)	0.22	84%	1.21 (0.89,1.63)	0.23	79%	1.13 (0.82,1.56)	0.45	76%
Ischemic stroke	1.50 (1.12,2.00)	0.28	84%	1.24 (0.89,1.73)	0.21	81%	1.01 (0.74,1.38)	0.95	68%
HWE (Y)	1.12 (0.93,1.34)	0.23	84%	1.19 (0.92,1.54)	0.18	79%	1.05 (0.82,1.35)	0.68	74%
Score≥8	0.99 (0.85,1.14)	0.85	73%	1.01 (0.90,1.15)	0.81	0%	0.95 (0.70,1.29)	0.74	83%

*p*, a *p*-value of combined effect; CI: confidence interval.

We also performed a meta-analysis to detect any association between males and females; however, only the recessive model (OR = 1.35, 95% CI = 1.07) identified a risk of hypertension among females.

**Figure 3:**
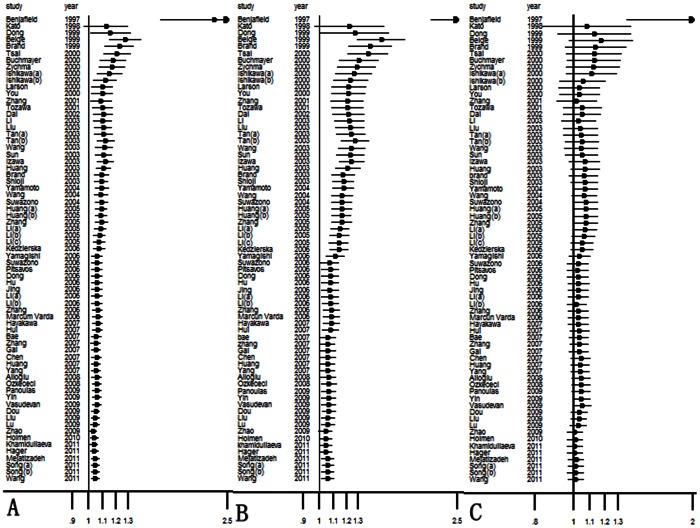
A cumulative plot by publication year for (A) the allelic model, (B) the dominant model, and (C) the recessive model. The ORs and associated 95% CIs became more stable over time. The study by Benjafield et al. [41] was the first report to show a significant association between the *GNB3* C825T polymorphism and the risk of hypertension, and this study likely influenced the overall estimation.

In the cumulative meta-analysis by year of publication, the ORs and 95% CIs became more stable (Figure 3). Study by Benjafield et al. [41] was the first publication to report a significant association between the *GNB3* C825T polymorphism and hypertension and triggered the identification of subsequent related studies that tried to replicate the initial results. In the allelic, dominant, and recessive models, the study by Benjafield et al. [41] was the most influential and made the overall estimation more significant in the present cumulative meta-analysis. After the study by Dou et al. [42] was included, the overall estimation became more accurate for the larger sample size.

### Subgroup Analysis

To further clarify heterogeneity among the studies, we performed subgroup analysis. Regarding the hypertension study population, the status of the HWE and the source of control had a critical role in heterogeneity (detailed data is presented in Table 4). Interestingly, only the allelic model, which was not consistent with the HWE, yielded a marginally significant risk of hypertension (OR = 1.18, 95% CI = 1.06–1.33), but no evidence of an association was found in the source of the control studies (controls were population-based or hospital-based).

Only one publication regarding Caucasians was screened in an analysis of the association between the *GNB3* C825T polymorphism and stroke, and all of the control sources were population-based, thus we did not perform subgroup analysis by ethnicity. Similarly, there were only two studies regarding an African population and hypertension, further indicating that subgroup analysis by ethnicity was to be avoided.

### Sensitivity Analysis

To further strengthen the confidence of the results of this meta-analysis, sensitivity analysis was conducted by limiting the included studies with NOS scores ≥8 or restricted analysis on hypertension populations according to the HWE and without other diseases or only included Asian and/or Caucasian populations. All comparative models found no association with hypertension, which suggested that the T allele or TT genotype may not be a risk factor for hypertension (detailed data is presented in Table 4). Importantly, the sensitivity analysis results were slightly out of agreement with those of the initial analysis; therefore, the results should be interpreted cautiously.

As to the association of stroke, when we restricted the analyses by limiting the included studies according to the HWE, the recalculated pooled OR values did not alter the initial results, suggesting that the TT genotype or T allele was not a risk factor of stroke (detailed data are presented in Table 5). Similarly, when we evaluated the ischemic stroke population or Asian population, no evidence of statistical association was obtained.

### Publication Bias

**Figure 4:**
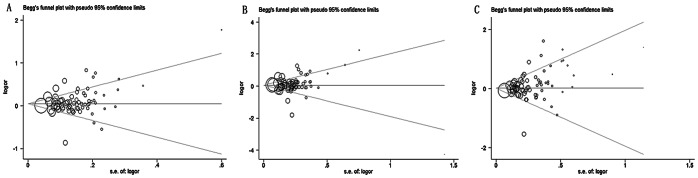
Funnel plots for the *GNB3* C825T polymorphism and its association with hypertension. (A) the allelic model (T allele vs. C allele, *p* = 0.150), (B) the dominant model (TT+CT vs. CC, *p = *0.565), and (C) the recessive model (TT vs. CT+CC, *p = *0.043). The funnel plots should be symmetrical when no publication bias occurs; however, the funnel plot of the recessive model was asymmetrical (*p* = 0.043), suggesting publication bias. The other two were symmetrical (*p = *0.150 and 0.565, respectively). SE, standard error; OR, odds ratio.

**Figure 5:**
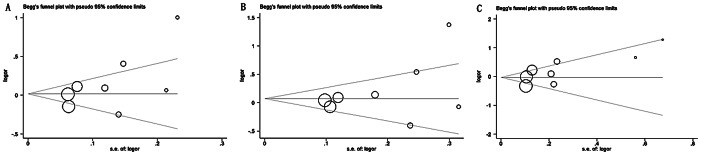
Funnel plots for the *GNB3* C825T polymorphism and its association with stroke. (A) the allelic model (T allele vs. C allele, *p = *0.145), (B) the dominant model (TT+CT vs. CC, *p = *0.281), and (C) the recessive model (TT vs. CT+CC, *p = *0.116). The funnel plots should be symmetrical when no publication bias occurs. No evidence of publication bias was detected in the three models. SE, standard error; OR, odds ratio.

Funnel plots were constructed and the Egger's test was performed to assess publication bias of the studies. Funnel plots should be symmetrical when no publication bias exists (Figures 4 and 5). Regarding the hypertension population, only the recessive model displayed an asymmetric funnel plot, while the Egger's regression test confirmed the presence of moderate publication bias (*p* = 0.043). No statistical evidence of publication bias was identified regarding the *GNB3* C825T polymorphism and its association with stroke.

## Discussion

Stroke is a significant event that leads to increased mortality and morbidity and hypertensive individuals reportedly have a greater incidence of stroke than normotensive individuals. Genetic factors as well as obesity, high sodium intake, physical inactivity, low potassium diets, and alcohol consumption contribute to the occurrence of hypertension, and essential hypertension status may play a role in the etiology of stroke either through effects on blood pressure levels or through separate pathways [11], [43]. The established relationship between hypertension and stroke suggested that these disorders may have at least some genes in common. Recently, several studies reported that the *GNB3* 825T polymorphism was associated with an increased risk of hypertension, obesity, metabolic syndrome, atherosclerosis, and diabetes mellitus. Besides, the *GNB3* 825T allele was found to significantly increase the risk of clinical ischemic stroke in Caucasians, but not subclinical cerebral infarct [12], [44]. However, the present meta-analysis was designed to confirm the association between the *GNB3* C825T polymorphism and essential hypertension or stroke.

Overall, our meta-analytical results showed that the *GNB3* 825T allele had a weak association with essential hypertension. However, after we restricted the studies according to the HWE and included only those without other diseases, such as diabetes and myocardial infarction, all of the compared models failed to identify an association between the *GNB3* 825T allele and hypertension. Similarly, when we performed sensitivity analysis with the inclusion criteria of “Asian” or “Caucasian,” no evidence of an association was obtained, which might be due to heterogeneity between the studies. Besides, the funnel plot was asymmetric in the recessive model for *p* = 0.043, so publication bias must also be considered. In addition, our results were consistent with those reported in previous studies [45], [46], but were slightly less discrepant with others [47], which might have resulted from the greater number of studies included in our meta-analysis. However, there were only two studies concerning an African population, thus a larger sample size is needed to further address the relationship between the *GNB3* C825T polymorphism and essential hypertension in Africans.

Interestingly, the *GNB3* C825T polymorphism was not associated with stroke. When we retrieved studies on ischemic stroke cases or limited the studies according to the HWE or an NOS score of ≥8, similar results were obtained, suggesting that our initial results were reliable and in line with most of the included studies. But, considering that most of the included stroke patients were Asian, our results cannot be directly used to extrapolate a correlation between the GNB3 c825T polymorphism and stroke in Caucasians, Africans, or other ethnicities.

In addition, we tested the T allele frequency in controls (hypertensive population) (Table 1), and found that there was statistical significance between Asian, Caucasian, and African groups (*p* = 0.0001). This result was in agreement with a previous study [48] that reported varied frequencies of the T allele among different ethnic groups, in which the highest rate occurred in Africans (T = 79%), followed by Asians (T = 46%), and then Caucasians (T = 33%). However, no statistical significance was found between males and females (*p* = 0.337). Therefore, it is likely that a higher T allele frequency is not necessarily indicative of an increased occurrence of hypertension.

Generally, the *GNB3* 825T allele was only slightly associated with an increased risk of essential hypertension compared to non-carriers. But, the *GNB3* C825T polymorphism failed to contribute to the risk of stroke, thus it was clear that the polymorphism contributed to hypertension and stroke differently. Therefore, gene-gene interactions should be taken into consideration. Until now, >500 candidate genes for hypertension have been suggested from a variety of genetic studies, and this number continues to increase [1], but not all of these genes were associated with an increased risk of stroke. Distribution of the C825T genotypes varies greatly in different ethnicities and the frequency of the T allele is highest in Africans, lowest in Caucasians, and intermediate in Asians. However, the CC genotype is rare in Africans and the distribution of East Asian genotypes is roughly 25% TT, 50% TC, and 25% CC [4]. Thus, individuals from different ethnicities may develop cardiovascular disorders, such as hypertension or stroke, which more or less differ in pathogenesis/pathophysiology of a given disorder due to different genetic backgrounds. In our meta-analysis, individual studies on Africans, Asians, and other ethnicities were deficient; therefore, additional evidence regarding the correlation of the *GNB3* C825T polymorphism with hypertension or stroke is required. The interactions between environmental and genetic factors constitute a key issue in the pathogenesis of hypertension and stroke. Most of the susceptibility genes for common diseases such as hypertension do not have a strong primary etiological role in disease predisposition, but rather code response elements to exogenous environmental factors. Therefore, a genetic marker may have only a modest affect on calculating risk in individuals who minimize exposure to environmental factors, but a major effect in individuals exposed to high-risk environment factors [49]. Young et al. [50] reported that latitude was an ecological factor that affected blood pressure via temperature and humidity. Likewise, *GNB3* presents a number of functional alleles that influence hypertension susceptibility. Therefore, those populations that have a high prevalence of the *GNB3* 825T allele also have a higher prevalence of heat-adapted alleles at other SNPs. Physical inactivity, increased body mass, obesity, and smoking may also influence the risk of hypertension and stroke differently.

In addition to race, gender also seems to be an important risk factor for adverse cardiovascular events, such as hypertension and stroke. Suwazono et al. [51] reported that the 825T allele was an independent risk factor for hypertension in Japanese females, whereas Beige et al. [52] found that the T allele in males was associated with higher blood pressure. In our analysis, the *GNB3* TT genotype was marginally associated with hypertension among females, and no evidence of an association with hypertension was found in males. In the cumulative meta-analysis, three models showed evidence of a non-association between *GNB3* alleles and hypertension or stroke. Two primary causes may account for this discrepancy. First, females have dominant parasympathetic and subordinate sympathetic activities compared to males, and, secondly, estrogen plays an important role in gender-related differences in the autonomic nervous system [51]. Thus, it seems that different automatic functions between genders altered the association of the *GNB3* 825T allele with hypertension.

Some limitations of the present meta-analysis should be considered. Firstly, all of the included studies mostly involved Caucasians and Asians, thus studies on other ethnic populations are needed. Secondly, all of the included studies were case-controlled and all of the cases involved survivors of hypertension and stroke. Finally, the number of stroke cases were limited and had relatively weak statistical power to detect potential risks of the *GNB3* C825T polymorphism. Thus, more population-based studies with large sample sizes are required. Despite these limitations, this meta-analysis was designed to overcome the limitations of individual studies, thus the results should be more reliable. Since the *GNB3* C825T polymorphism appears to be a useful marker to predict the relative risk of diseases, such as hypertension and stroke, this meta-analysis is better suited in a preventive aspect to identify certain genotypes that will be most likely to benefit from pharmacological interventions.

In summary, the overall analysis of available evidence suggested that the *GNB3* 825T allele may be a good indicator of hypertension; however, it had no association with hypertension in Asians and Caucasians and there was lack of evidence to support an association with stroke in Asians. Therefore, multiethnic studies with much larger sample-sizes are required to better evaluate the association between the *GNB3* C825T polymorphism and hypertension or stroke.

## Supporting Information

Appendix S1 **PRISMA Checklist.**
(DOC)
