# Supplementary material for: The C825T Polymorphism of the G-Protein β3 Subunit Gene and Its Association with Hypertension and Stroke: An Updated Meta-Analysis
Source: PLoS One. 2013 Jun 14;8(6):e65863. doi: 10.1371/journal.pone.0065863 (PMC3682991; doi:10.1371/journal.pone.0065863)
Supplement: Appendix S1 — PRISMA Checklist. (DOC) [file pone.0065863.s001.doc]

| **Section/topic** | **#** | **Checklist item** | **Reported on page #** |
| --- | --- | --- | --- |
| **TITLE** | | |  |
| Title | 1 | The C825T polymorphism of the G-protein β3 subunit gene and its association with hypertension and stroke: an updated meta-analysis | Page 1 |
| **ABSTRACT** | | |  |
| Structured summary | 2 | Provide a structured summary including, as applicable: background; objectives; data sources; study eligibility criteria, participants, and interventions; study appraisal and synthesis methods; results; limitations; conclusions and implications of key findings; systematic review registration number.  Background and Objective: Several epidemiological studies have evaluated the association between the GNB3 C825T polymorphism and hypertension or stroke, but the results were inconsistent; therefore, we performed a meta-analysis to clarify these discrepancies.  Methods: We systematically searched the PubMed, Embase, Web of Science, CNKI, and CBM databases, and manually searched reference lists of relevant papers, meeting abstracts, and relevant journals. Pooled odds ratios (ORs) and 95% confidence intervals (CIs) were calculated for dominant, recessive, and allelic models. A fixed or random effects model was separately adopted depending on study heterogeneity. Subgroup and sensitivity analyses were performed to detect study heterogeneity and examine result stability, respectively. Publication bias was tested using funnel plots, the Egger's regression test, and Begg's test.  Results: We screened 66 studies regarding hypertension and eight concerning stroke. A combined analysis showed that only the allelic model found a marginal association with hypertension (OR = 1.07, 95% CI = 1.01–1.13) and female gender (OR = 1.11, 95% CI = 0.99–1.24). However, no comparison models found an association with stroke (allelic model: OR = 1.11, 95% CI = 0.94–1.32; dominant model: OR = 1.16, 95% CI = 0.92–1.48; and recessive model: OR = 1.05, 95% CI = 0.97–1.14). Sensitivity analysis suggested that all models did not yield a relationship to hypertension or stroke among Asians. Besides, there was a lack of statistical association with hypertension in Caucasians, which maybe due to a small sample size. When we restricted the included studies to normal populations according to the Hardy–Weinberg equilibrium, no association was found.  Conclusions: There was no evidence indicating that the C825T allele or TT genotype was associated with hypertension or stroke in Asians or hypertension in Caucasians. However, further studies regarding Africans and other ethnicities are needed to identify further correlations. | Page 1-2 |
| **INTRODUCTION** | | |  |
| Rationale | 3 | Describe the rationale for the review in the context of what is already known.  Hypertension is a major risk factor of stroke, cardiovascular disease, and end-stage renal disease and affects about 1 billion adults worldwide. Stroke is a primary contributor to long-term adult disability and the third most common cause of death in developed countries. Blood pressure-lowering therapies are viewed as protective measures against the risk of hypertension and stroke, but both genetic and lifestyle factors are likely involved in their development.  G proteins consist of α, β, and γ subunits are key determinants of specific and temporal characteristics of many signaling processes. Activation of a G protein-coupled receptor results in an exchange of guanosine triphosphate for guanosine diphosphate followed by dissociation of the α subunit from the βγ complex. Different α subunits can then regulate a large variety of intracellular signaling cascades. The α subunit and βγ complex then reassemble as a heterotrimer available for a new activation cycle. Alterations in G protein signaling can induce multiple disorders.  The GNB3 gene encodes the Gβ3 subunit of heterotrimeric G proteins and is located on chromosome 12p13 and comprises 11 exons and 10 introns. A polymorphism (C825T, rs5433) was found to be associated with a shortened splice variant of the Gβ3 protein that gives rise to enhanced signal transduction via pertussis toxin-sensitive G proteins. Differential G protein activities associated with the C825T SNP did not result from different transcript amounts associated with specific GNB3 genotypes.  Several epidemiological studies have shown an association between the GNB3 C825T allele and other features of metabolic syndrome, including obesity, insulin resistance, changes in autonomic nervous function, and dyslipidemia. This polymorphism has also been identified in hypertension, stroke, Alzheimer’s disease, sudden death, tumor progression, and as a genetic marker for drug responses to diuretics, antidepressants, sildenafil, clonidine, and sibutramine. | Page 2-3 |
| Objectives | 4 | Provide an explicit statement of questions being addressed with reference to participants, interventions, comparisons, outcomes, and study design (PICOS).  Draw a more authentic association between the GNB3 C825T polymorphism and hypertension or stroke.  Participants: population suffering from hypertension or stroke; interventions: testing the genotype of GNB3; comparisons: the association between GNB3 C825T and hypertension or stroke; outcomes: the risk for hypertension or stroke; study design: case-control study. | Not available |
| METHODS | | |  |
| Protocol and registration | 5 | Indicate if a review protocol exists, if and where it can be accessed (e.g., Web address), and, if available, provide registration information including registration number.  There is not a review protocol exists. | Not available |
| Eligibility criteria | 6 | Specify study characteristics (e.g., PICOS, length of follow-up) and report characteristics (e.g., years considered, language, publication status) used as criteria for eligibility, giving rationale.  (1) population-based or hospital-based case-control studies regarding the relationship between the GNB3 C825T polymorphism and essential hypertension or stroke; (2) sufficient data on genotypic and allelic frequencies to determine an odds ratio (OR) with a 95% confidence interval (CI). If multiple publications reported the same or overlapping data, the most recent or largest population or complete study was included in this meta-analysis as described by Little et al.; (3) to avoid local literature bias, publications in both Chinese and English were considered; (4) studies with related clinical characteristics were limited to those using human subjects; (5) articles regarding cases compounded with other diseases, such as diabetes mellitus and myocardial infarction, were also included; and (6) if patient blood pressure was measured casually or ambulatory (24 h), the latter were used; (6) hypertension was defined as mean casual blood pressure ≥ 140/90mmHg or mean ambulatory blood pressure > 134/79mmHg. | Page 4-5 |
| Information sources | 7 | Describe all information sources (e.g., databases with dates of coverage, contact with study authors to identify additional studies) in the search and date last searched.  We comprehensively searched for related papers in the following electronic databases: PubMed (up to Nov 2012), Embase (1996 to Nov 2012), Web of Science (2003 to Nov 2012), CBM (China Biology Medicine, 1978 to Jul 2012) and CNKI (China National Knowledge Infrastructure, 1999 to Nov 2012). | Page 4 |
| Search | 8 | Present full electronic search strategy for at least one database, including any limits used, such that it could be repeated.  We have used various keywords, including “hypertension,” “stroke,” “cerebral hemorrhage,” “cerebrovascular disorder,” “cerebrovascular disease,” “mutation,” “variant,” “polymorphism,” “ischemic stroke,” “GNB3,” “G protein beta,” and “G-beta.” Then, we manually searched the relevant journals and co-authors listed in the included studies to find additional studies. Reference lists of all retrieved publications were also checked for missing information. Meeting abstracts, which were previously shown to influence meta-analytical results, were also scrutinized. All relevant articles were initially scanned on the basis of title, keywords, and abstract. If not, the full text was obtained for further evaluation. The literature retrieval was performed independently by three investigators (LG, LLZ, and BZ) and discrepancies were resolved by reaching a consensus among the investigators. If a consensus could not be established, a fourth reviewer (JCL) was consulted to resolve the discrepancy. | Page 4 |
| Study selection | 9 | State the process for selecting studies (i.e., screening, eligibility, included in systematic review, and, if applicable, included in the meta-analysis).  Screening the relevant studies based on title and abstract. Next, review articles, comment and meeting abstracts without sufficient data were excluded. Finally, reading the full-text, wrong model, non-hypertension, non-human samples or non-stroke, no outcome of interest, studies without sufficient data or with duplicate data were excluded. | Page 4 |
| Data collection process | 10 | Describe method of data extraction from reports (e.g., piloted forms, independently, in duplicate) and any processes for obtaining and confirming data from investigators.  Data were extracted from each study by three investigators (LG, LLZ, and BZ) independently following the above-mentioned inclusion criteria. Discordance was resolved by discussion, or another viewer (JCL) was involved. | Page 5 |
| Data items | 11 | List and define all variables for which data were sought (e.g., PICOS, funding sources) and any assumptions and simplifications made.  We sought the data based on the genotypes counts in the cases and controls. | Page 4-5 |
| Risk of bias in individual studies | 12 | Describe methods used for assessing risk of bias of individual studies (including specification of whether this was done at the study or outcome level), and how this information is to be used in any data synthesis.  In our meta-analysis, all the included studies were case-controls ones. We did not describe adequate sequence generation, allocation concealment, blinding, incomplete outcome data addressed, free of selective reporting or free of other bias. | Page 4 |
| Summary measures | 13 | State the principal summary measures (e.g., risk ratio, difference in means).  The strength of association of the GNB3 C825T polymorphism with hypertension or stroke was measured by calculating summary ORs with corresponding 95% CIs for the dominant model (TT+CT vs. CC), recessive model (TT vs. CT+CC), and allelic model (T allele vs. C allele), respectively. | Page 5-6 |
| Synthesis of results | 14 | Describe the methods of handling data and combining results of studies, if done, including measures of consistency (e.g., I2) for each meta-analysis.  Heterogeneity between the studies was analyzed using the Cochran’s Q test and the I2 statistic (range, 0–100%) . If the results of the Q test was p < 0.1 and the measure of I2 was > 50%, indicating significant heterogeneity between studies, the ORs were pooled using a fixed effects Mantel–Haenszel method , otherwise the DerSimonian and Laird random effects model was adopted. | Page 6 |

Page 1 of 2

| Section/topic | # | Checklist item | Reported on page # |
| --- | --- | --- | --- |
| Risk of bias across studies | 15 | Specify any assessment of risk of bias that may affect the cumulative evidence (e.g., publication bias, selective reporting within studies).  Publication bias was assessed by Egger's regression test and Begg's test. In order to avoid local literature bias, publication language in Chinese and in English were also considered. To avoid selection bias, no study was rejected because of poor quality parameters. | Page 6 |
| Additional analyses | 16 | Describe methods of additional analyses (e.g., sensitivity or subgroup analyses, meta-regression), if done, indicating which were pre-specified.  A Galbraith plot was employed to detect potential sources of heterogeneity. To further detect heterogeneity, subgroup analyses were performed using the status of the HWE (yes or no) or the control source. Sensitivity analysis was conducted by limiting the meta-analysis to high quality studies (NOS score ≥ 8). We also reconducted the analyses by limiting the studies according to the HWE and excluding those that included myocardial infarction, obesity, or diabetes mellitus in the cases or controls. Cumulative meta-analysis was performed to identify the influence of the first published study on the subsequent publications concerning the relationship between the GNB3 C825T polymorphism and hypertension, and to estimate the combined estimate over time. Subgroup analyses and sensitivity analysis were pre-specified. | Page 6 |
| RESULTS | | |  |
| Study selection | 17 | Give numbers of studies screened, assessed for eligibility, and included in the review, with reasons for exclusions at each stage, ideally with a flow diagram.  (1)For hypertension: 996 potentially relevant studies were screened(PubMed 221, Embase 269, Web of Science 411, CBM 36, CNKI 59). Review articles (n=74), comment (n=3), meeting abstracts (n=12) were excluded. Besides, wrong model, non-hypertension, non-human samples, no outcome of interest (n=820) were also excluded, and studies without sufficient data or with duplicate data (n=21) were exclude. In the final, 66 studies were included in this meta-analysis.  (2)For stroke: 121 potentially relevant studies were screened(PubMed 39, Embase 17, Web of Science 55, CBM 8, CNKI 10). Review articles (n=11), studies without sufficient data or with duplicate data (n=2) as well as non-stroke, no outcome of interest, no controls or the same studies included in different database (n=100) were excluded. In the final, 8 studies were included in this meta-analysis. | Page 7 and Fig. 1 |
| Study characteristics | 18 | For each study, present characteristics for which data were extracted (e.g., study size, PICOS, follow-up period) and provide the citations.  The following data was collected from each of the selected studies: surname of the first author, year of publication, country of origin, population ethnicity, source of control, T allele frequency in controls, genotype variance in the cases and controls, and the Hardy–Weinberg equilibrium (HWE) using the χ2 test. A p-value < 0.05 of the HWE was considered statistically significant. | Page 5 |
| Risk of bias within studies | 19 | Present data on risk of bias of each study and, if available, any outcome level assessment (see item 12).  We did not take considerations for presentation of risk of bias assessments of each study. | Not available. |
| Results of individual studies | 20 | For all outcomes considered (benefits or harms), present, for each study: (a) simple summary data for each intervention group (b) effect estimates and confidence intervals, ideally with a forest plot.  The results of this meta-analysis were presented in Tables 4 and 5. And we have only presented the forest plots for the association between the GNB3 C825T polymorphism with stroke, each of the relevant studies’ effect estimates and confidence intervals could be identified in these three plots. | Page 23 |
| Synthesis of results | 21 | Present the main results of the review. If meta-analyses are done, include for each, confidence intervals and measures of consistency.  A significant overall association between the GNB3 C825T polymorphism and the risk of hypertension was only detected in the allelic model (OR = 1.07, 95% CI = 1.01–1.13). However, none of the comparison models found an association between the GNB3 C825T polymorphism and stroke (allelic model: OR = 1.11, 95% CI = 0.94–1.32; dominant model: OR = 1.16, 95% CI = 0.92–1.48; and recessive model: OR = 1.05, 95% CI = 0.97–1.14, respectively) (Figure 2). | Page 8 |
| Risk of bias across studies | 22 | Present results of any assessment of risk of bias across studies (see Item 15).  Regarding the hypertension population, only the recessive model displayed an asymmetric funnel plot, while the Egger's regression test confirmed the presence of moderate publication bias (p = 0.043). No statistical evidence of publication bias was identified regarding the GNB3 C825T polymorphism and its association with stroke. | Page 9-10 |
| Additional analysis | 23 | Give results of additional analyses, if done (e.g., sensitivity or subgroup analyses, meta-regression [see Item 16]).  Regarding the hypertension study population, the allelic model, which was not consistent with the HWE, yielded a marginally significant risk of hypertension (OR = 1.18, 95% CI = 1.06–1.33), but no evidence of an association was found in the source of control studies (controls were population-based or hospital-based).  Sensitivity analysis was conducted by limiting the included studies with NOS scores ≥ 8 or restricted analysis on hypertension populations according to the HWE and without other diseases or only included Asian and/or Caucasian populations. All comparative models found no association with hypertension. As to the association of stroke, when we restricted the analyses by limiting the included studies according to the HWE, the recalculated pooled OR values did not alter the initial results.  In the cumulative meta-analysis by year of publication, the ORs and 95% CIs became more stable. Study by Benjafield et al. was the first publication to report a significant association between the GNB3 C825T polymorphism and hypertension and triggered the identification of subsequent related studies that tried to replicate the initial results. In the allelic, dominant, and recessive models, the same study was the most influential and made the overall estimation more significant in the present cumulative meta-analysis. After the study by Dou et al. was included, the overall estimation became more correct for the larger sample size. | Page 8-9 |
| DISCUSSION | | |  |
| Summary of evidence | 24 | Summarize the main findings including the strength of evidence for each main outcome; consider their relevance to key groups (e.g., healthcare providers, users, and policy makers).  The frequency of T allele varies in different ethnic groups (highest in blacks, lowest in white subjects, and intermediate in Asians (as for hypertension). Our meta-analysis was designed to overcome the limitations of individual studies, thus the results should be more reliable. | Page 13 |
| Limitations | 25 | Discuss limitations at study and outcome level (e.g., risk of bias), and at review-level (e.g., incomplete retrieval of identified research, reporting bias).  First, all of the included studies mostly involved Caucasians and Asians, thus studies on other ethnic populations are needed. Secondly, all of the included studies were case-controlled and all of the cases involved survivors of hypertension and stroke. Finally, the number of stroke cases were limited and had relatively weak statistical power to detect potential risks of the GNB3 C825T polymorphism. | Page 13 |
| Conclusions | 26 | Provide a general interpretation of the results in the context of other evidence, and implications for future research.  In summary, the overall analysis of available evidence suggested that the GNB3 825T allele may be a good indicator of hypertension; however, it had no association with hypertension in Asians and Caucasians and there was lack of evidence to support an association with stroke in Asians. Therefore, multiethnic studies with much larger sample-sizes are required to better evaluate the association of the GNB3 C825T polymorphism to hypertension or stroke. | Page 13 |
| FUNDING | | |  |
| Funding | 27 | Describe sources of funding for the systematic review and other support (e.g., supply of data); role of funders for the systematic review.  This work was supported by grants from the National Natural science Foundation of China (No. 81271282, No. 30970998), a grant from the Chongqing Natural Science Foundation (No. CSTC2011BB5031). | Page 13 |

*From:*  Moher D, Liberati A, Tetzlaff J, Altman DG, The PRISMA Group (2009). Preferred Reporting Items for Systematic Reviews and Meta-Analyses: The PRISMA Statement. PLoS Med 6(6): e1000097. doi:10.1371/journal.pmed1000097

For more information, visit: **www.prisma-statement.org**.

Page 2 of 2
